# RNA inverse folding can be solved in linear time for structures without isolated stacks or base pairs

**DOI:** 10.1186/s13015-025-00278-6

**Published:** 2025-10-24

**Authors:** Théo Boury, Samuel Gardelle, Laurent Bulteau, Yann Ponty

**Affiliations:** 1 Laboratoire d’Informatique de l’Ecole Polytechnique (LIX CNRS UMR 7161), Institut Polytechnique de Paris, 1 Rue Honoré d’Estienne d’Orves, 91120 Palaiseau, France; 2 Laboratoire d’Informatique Gaspard Monge (LIGM CNRS UMR 8049), Université Gustave Eiffel, 5 Boulevard Descartes – Champs sur Marne, 77454 Marne La Vallée, France

**Keywords:** RNA structure, String design, Parameterized complexity, Uniform sampling

## Abstract

Inverse folding is a classic instance of negative RNA design which consists in finding a sequence that uniquely folds into a target secondary structure with respect to energy minimization. A breakthrough result of Bonnet *et al.* shows that, even in simple base pairs-based (BP) models, the decision version of a mildly constrained version of inverse folding is NP-hard. In this work, we show that inverse folding can be solved in linear time for a large collection of targets, including every structure that contains no isolated BP and no isolated stack (or, equivalently, when all helices consist of $3^{+}$ base pairs). For structures featuring shorter helices, our linear algorithm is no longer guaranteed to produce a solution, but still does so for a large proportion of instances. Our approach introduces a notion of modulo *m*-separability, generalizing a property pioneered by Hales *et al*. Separability is a sufficient condition for the existence of a solution to the inverse folding problem. We show that, for any input secondary structure of length *n*, a modulo *m*-separated sequence can be produced in time $\mathcal {O}(n\,m\, 2^m)$ anytime such a sequence exists. Meanwhile, we show that any structure consisting of $3^{+}$ base pairs is either trivially non-designable, or always admits a modulo-2 separated solution. Solution sequences can thus be produced in linear time, and even be uniformly generated within the set of modulo-2 separable sequences.

## Introduction

RNA inverse folding is a fascinating algorithmic problem which, given a target secondary structure *T*, consists of designing one or several sequences, all of which should uniquely fold into the target *T* according to a reference folding prediction algorithm. Considering a folding prediction algorithm as a mathematical function $\Phi : \{A,C,G,U\}^\star \rightarrow \mathcal {S}\cup \{\perp \}$ mapping an RNA sequence to a unique predicted structure (or $\perp$ if equally likely alternatives exist), inverse folding can be abstracted as the search for a preimage $w\in \Phi ^{-1}(T)$ of the target structure *T*. This naturally generalizes into a variety of design tasks which, given a predictive algorithm implementing a function $\Phi$, aim to create one or multiple instances predicted to behave in a certain way. Such a formulation is, in general, overly broad (e.g. it encompasses the concept of one-way functions in cryptography) to inspire reasonable hopes for a general solution. Still, a restriction of the inverse problem to certain types of computable functions/algorithms (e.g. amenable to dynamic programming) appears realistic and generally relevant to (synthetic) biology, yet poorly studied to this day.

In the specific case of RNA, despite being the object of substantial attention since its formal introduction in the early 1990s [1], the complexity of RNA inverse folding has remained elusive for almost three decades. A generalization of RNA inverse folding, including the energy model as part of the input, was shown to be NP-hard by Schnall-Levin et al. [2]. However, their reductions critically relied on (ab)using the energy model to encode a 3SAT instance, leaving the hardness of the problem largely open for a fixed energy model. The classic complexity of inverse folding was only settled, in 2020, when Bonnet et al. [3] finally showed the NP-hardness of RNA folding in a classic base pairs maximization setting. Such computational intractability (retrospectively) legitimizes a very large quantity of heuristic or exponential-time methods, based on local search [1, 4–7], bio-inspired metaheuristics [8–11], global sampling [12, 13], constraint programming [14, 15] and, more recently, neural networks-inspired generative models [16].

In parallel to complexity studies, Hales et al. [17] revisited the problem from a structural angle, attempting to characterize designable or undesignable families of secondary structures. The authors showed that saturated structures, having all positions paired, are designable if and only if their multiloop degrees do not exceed 4. They also introduced a notion of separability, a sufficient, yet not necessary in general, condition for a sequence to be a design for a given target. This notion allowed them to show that any target structure either features an occurrence of a locally-undesignable motif $\{m_{3\bullet },m_{5}\}$, or can always be transformed into a separable structure by adding at most one base pair per helix. More strikingly, they proposed linear-time algorithms for producing a single solution for each characterized class of designable structures, painting a – puzzling – contrasted picture of general hardness (as per Bonnet et al. [3]) and practical facility for inverse folding.

In this work, we further those studies and show that:

- While conceptually elegant, we show that separability unfortunately remains challenging: Finding a separated design for a given structure is NP-hard, even when restricted to structures avoiding isolated base pairs;
- Conversely, we prove that any structure with helices of length greater than 3 base pairs is either trivially not designable (i.e. contains $\{m_{3\bullet },m_{5}\}$), or separable. Moreover, if designable, a solution sequence can then be designed in linear-time. This constraint is relevant to the objectives of RNA design, as targeted secondary structures are typically stable and tend to avoid shorter – unstable – helices;
- To establish this result, we introduce of the concept of modulo *m*-separability, a refined version of separability, which coincides with general separability upon setting $m\ge n/2$. Deciding *m*-separability clearly remains NP-hard in general, but it can be solved (+ a solution sequence be produced) in time $\mathcal {O}(n\, 2^m)$ by a Fixed-Parameter Tractable (FPT) algorithm for *m*;
- We prove that this algorithm solves all instances of inverse folding with minimal helix lengths of 3 BPs when invoked with $m=2$ and, even in this restricted setting, many instances with shorter helices;
- We adapt our algorithm into a uniform random generator of separated designs, combining a mildly unambiguous dynamic programming scheme with a rejection strategy that achieves an average-case $\mathcal {O}(n\,m\,2^m)$ time complexity;
- Finally, we empirically observe that *m*-separated sequences often represent solutions for instances featuring isolated base pairs or stacks. Moreover, despite being only guaranteed to represent designs with respect to base pair maximization, are also likely to represent designs in the more realistic Turner energy model and, in a relaxed setting, are also superior than mere compatible sequences for multiloops of larger cardinalities. Finally, we observe that *m*-separated sequences seem to offer sufficient diversity to enable a control of the $\textsf {GC}\%$.

## Problem statement, definitions, and prior work

Algorithmically, RNA can be abstracted as a nucleotide sequence, i.e. a string $w\in \Sigma ^n$, $\Sigma :=\{\textsf {A},\textsf {C},\textsf {G},\textsf {U}\}$, where *n* denotes the length of $w$. Given a length *n*, a (non crossing/pseudoknot-free) secondary structure is a set $T\subset [1,n]^2$ consisting of base pairs such that:

- Each position in [1, *n*] is involved in at most one base pair;
- Base pairs in $T$ are pairwise non-crossing: $\forall (i,j)\ne (k,l) \in T$, $i<k$, either $i<k<l<j$ or $i<j<k<l$.
- Minimal distance in nucleotide number is parameterized by $\theta$ ($\theta$ set to 0 by default).

The set $\mathcal {S}_{w}$ of secondary structures compatible with an RNA sequence $w$ is defined as: ${\mathcal {S}}_{w}:= \left\{ \text {Secondary structure }T\mid \forall (i,j)\in T, \{w_i,w_j\} \in \left\{ \{\textsf {G},\textsf {C}\},\{\textsf {A},\textsf {U}\},\{\textsf {G},\textsf {U}\}\right\} \right\} .$

Without loss of generality, a secondary structure can be represented as a tree $T=(V(T), E(T))$, whose nodes $V(T)$ are in bijection with base pairs (internal nodes^1^) and unpaired regions (leaves), and whose edges represent the inclusion of base pairs. Given a node $v \in V(T)$, we denote by $\text {parent}(v)$ the parent of *v* in $T$, and by $\text {children}(v)$ the list of children of *v* in $T$. A *loop* is the subtree restricted to a node and its (direct) children. The tree is rooted in a special $\textsf {Root}$ node, associated with the whole sequence interval. An *helix* of length $\ell$ of the tree is a maximal path $v_1,\ldots , v_\ell$ of base pair nodes such that each $v_i$ with $i<\ell$ has a single child $v_{i+1}$ (no leaf attached). A helix of length 1 is an *isolated base pair*. A helix of length 2 is an *isolated stack*. We define $h_{\min }$ as the minimum length over all helices of $T$. As the target tree is always explicit and unmodified through proofs and algorithms we do not specify it explicitly in the notations.

RNA inverse folding considers a target secondary structure $T$, and constructs a sequence $\omega \in \Sigma ^n$ whose unique base-pair maximizing secondary structure is $T$.

### Problem 1

Inverse_Folding_BP_

**Input:** Target secondary structure $T$, sequence length *n*

**Output:** Sequence $w\in \Sigma ^n$ satisfying both:

- Compatibility with target structure: $T\in \mathcal {S}_{w}$;
- Uniqueness of the target as the optimal fold for the sequence:
  $$\forall T' \in \mathcal {S}_{w}, T'\ne T, |T'|<|T|.$$

or $\perp$ if no such sequence exists.

Nevertheless, ${\textsc{Inverse\_Folding}}_\text {BP}$, mildly extended to allow further restrictions on individual sequence positions, was shown to be NP-hard by Bonnet et al. [3]. (The used restriction requires the inclusion of some constraints of the form “nucleotide *i* must be labeled by the base letter *b*”)

**Fig. 1:**
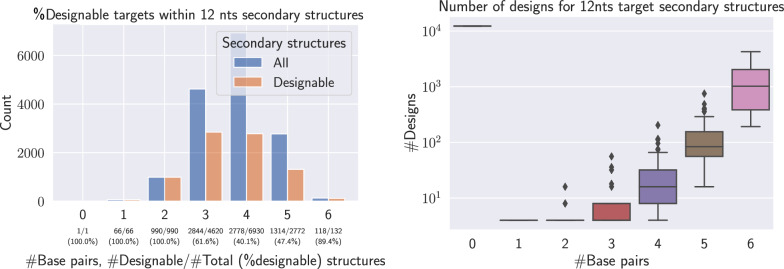
Exhaustive designability analysis of 12nts RNA sequences/structures. (Left) For a minimum base pair span of $\theta =0$, there exists 15 511 sary structures over 12 nucleotides, of which little over half (8 111) admits at least a solution to the inverse folding problem. (Right) The number of valid solutions varies substantially between targets and appears to depend on the number of base pairs. Overall, out of the 16 777 216 RNA sequences of length 12, only 399 348 ($\approx 2.4\%$) represent a valid design for some structure

A sequence is called a design for a structure $T$ if it represents a solution to the inverse folding problem for the input $T$. Note that the uniqueness condition can be tested in polynomial time using a variant of the Nussinov algorithm [17, 18]. In addition to showing that ${\textsc{Inverse\_Folding}}_\text {BP}$ is in NP, such an algorithm enables, for moderate sequence lengths, a systematic folding of all sequences in order to characterize the set of structures admitting a solution. For instance, Fig. 1 shows that, while only 2.4% of RNA sequences of length 12 represent a design for some target, roughly half of the secondary structure admits at least one solution sequence, and $\approx 49$ on average, for the inverse folding problem.

**Fig. 2:**
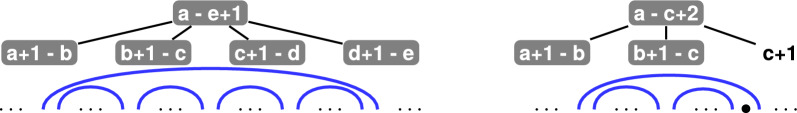
Forbidden motifs. Motifs $m_{5}$ (left) and $m_{3\bullet }$ (right), both shown as a tree (with *a*, *b*, *c* *d*, *e* arbitrary integers) and as nested base-pairs. Note that the relative order of the children base-pairs and the leaf in the $m_{3\bullet }$ pattern is irrelevant. Any assignment of base pair letters (either matching a proper coloring of the tree or not) leads to a possible local rerooting of at least two base pairs yielding an alternative thus making the structure undesignable [17]. Note that any such undesignable structure remains undesignable upon permuting children, or inserting new ones

**Fig. 3:**
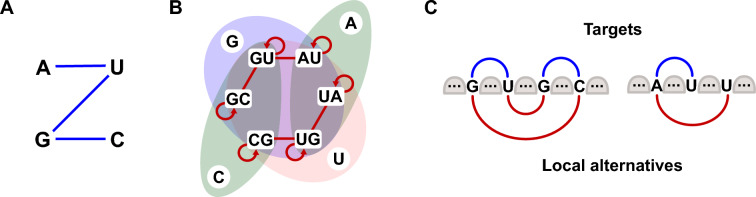
Local design rules. Base pair compatibility graph (**A**) and incompatibility graph for base pairs and unpaired nucleotides occurring within a loop (**B**): Connected base pairs, when jointly occurring within a loop of the target structure, can refold to form a local, an alternative structure having same number of base pairs as the target (**C**, left). Unpaired nucleotides may also interfere with some ($\textsf {A}$ or $\textsf {C}$) or every ($\textsf {G}$ or $\textsf {U}$) base pairs, leading to local alternatives (C, right)

We remind that, as noted by Halès et al. [17], two key motifs are not designable in a *base pair maximization* setting, see Fig. 2:

- The $m_{5}$ motif consists of 5 base pairs occurring on the same loop (not counting the Root). No sequence can be designed for such a motif, since exposing 5 base pairs on a loop always allows for local refolding to have the same number of base pairs. This follows from the inspection of Fig. 3, where the largest set of mutually compatible base pairs clearly has cardinality 4;
- The $m_{3\bullet }$ motif consists of 3 base pairs (excluding the Root) and one unpaired position. Indeed, as shown in Fig. 3, the presence of an unpaired nucleotide either forbids the co-occurrence of any adjacent base pair (G or U), or only allows three (C or A). Since at most two of those base pairs can co-occur in a successful loop design, $m_{3\bullet }$ is not designable.

Any occurrence of these structures (or any other undesignable structure [19]) as a subgraph of an instance makes the instance undesignable. In particular, the presence of a node with 4+ base-pair children, or 3+ children including two base-pairs and a leaf, leads to an undesignable structure.

### Inverse folding as a tree coloring problem

We start by reminding the coloring framework introduced by Halès et al. [17].

#### Definition 1

(Coloring) A coloring of a (secondary structure) tree $T$ is a function  associating a color to each node (except the root and the leaves which always get $\varnothing$).

A coloring of a tree *T* typically induces multiple RNA sequences that are compatible with, but not guaranteed to fold into, the given secondary structure through letters assignment rules. Namely, in any sequence $w$ derived from a coloring $\chi$, we have for each $(i,j)\in T$:

- If ;
- If ;
- If

For  nodes, the freedom in choosing $(\textsf {A}, \textsf {U})$ or $(\textsf {U},\textsf {A})$ depends on the context: the choice may be unconstrained (e.g. when isolated within a helix), or forced (e.g. when two gray nodes are involved in a multiloop or stack). However, this property will only impact the number of sequences associated with the coloring, but bears no consequence on the existence of a solution to ${\textsc{Inverse-Folding}}_\text {BP}$, since the problem asks for the production of a single sequence.

**Fig. 4:**
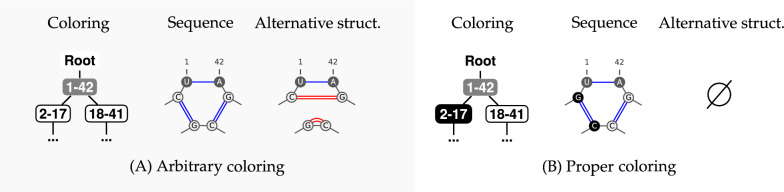
**A proper coloring is necessary towards design.** In (**A**), having two  children implies that the sequence derived from this coloring features a motif where $\textsf {G}$ and $\textsf {C}$ can reconfigure locally. In that case, they form an alternative structure that contains the same number of base pairs. Conversely, in (**B**), the proper coloring ensures that locally no alternative of equal (or better) energy exists by forcing some consecutive incompatibilities

Denote by $\overline{c}$ the inverse of a color *c*, defined as ,  and . Denote by $|C|_{c}$ the number of occurrences of color *c* in vector *C*.

#### Definition 2

(Proper Coloring) A coloring $\chi$ is proper when, for each node $v\in V(T)$, the vector of colors *C*, composed of the complementary color of the node concatenated with the colors of its children, respects the following constraints:
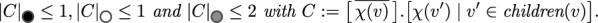


The use of the complementary color of *v* in *C* enables a compact definition: it forbids  and  to have respectively  and  children which would result in an alternative rerooting of the pairs. These conditions must also hold for the colorless $\textsf {Root}$, but with *C* being restricted to the colors of $\text {children}(\textsf {Root})$.

**Fig. 5:**
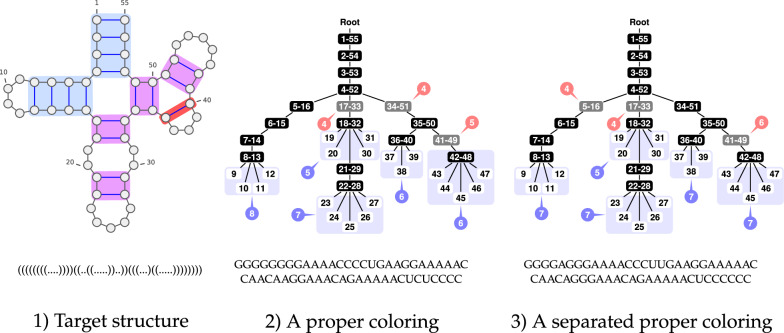
(1) 2D and dot-bracket representations of a secondary structure. Helices of sizes respectively 1 (isolated base pairs), 2 (isolated stacks) and more than 3 are represented in light red, purple and blue. (2) Same secondary structure as a tree. The tree is colored and levels are represented in red and blue bubbles. The coloring is proper but non-separated as the level of the leaf 19 is the same as the level of the  node 41-49. A non-separated coloring is not guaranteed to induce a design for its target, but may still do so, as is the case here. (3) Same secondary structure, colored in a separated (necessarily proper) manner. This coloring yields one or multiple designs (depending on the choice of AU or UA for  nodes). Notably, this coloring is 2-separated, as leaves and  nodes end up at odd and even levels respectively

In terms of RNA design, the proper condition is necessary for an associated sequence to be a solution to inverse folding. Indeed, any coloring that is not proper will be associated with sequences that can be locally reconfigured, this without losing any base pair (see Fig. 4 for local competing structures). (Fig. 5).

#### Definition 3

(Levels) Given a coloring $\chi$ of a tree $T$, the level $L: V(T) \rightarrow \mathbb {Z}$ of a node *v* is  where *p* denotes the color vector associated with the shortest node sequence from $\text {parent}(v)$ to $\textsf {Root}$.

On an RNA level, the concept of level helps categorize, and possibly control, the set of alternative structures to the target. Indeed, consider a sequence $w$ generated from a coloring $\chi$. First remark that, in order for an alternative structure to be competitive, every occurrence of $\textsf {C}$ must be paired. Whenever two positions *i* and *j* interact to form a base pair, it can be shown that the inner interval ]*i*, *j*[ interval contains $L(i)-L(j)$ more $\textsf {G}$ than $\textsf {C}$. Meanwhile the outermost interval $[1,i[\,\cup \, ]j,n]$ features the opposite imbalance ($L(i)-L(j)$ more $\textsf {C}$ than $\textsf {G}$). In other words, any structure that contains a base pair $(i,j)\notin T$ already has $2\times |L(i)-L(j)|$ fewer base pairs than the target structure. Thus only structures made of pairs (*i*, *j*) such that $L(i)=L(j)$ need to be considered as viable alternatives to $T$. This property can be exploited as a design principle, as formalized by the following property.

#### Definition 4

(Separated coloring) A coloring $\chi$ is separated for a target $T$ if and only if it is proper and the levels of -colored nodes and leaves do not overlap: 

This immediately suggests a design strategy that associates A to unpaired positions and assigns  and  colors such that  nodes end up as different levels as the leaves. Indeed, in this setting, Hales et al. [17] showed that the proper coloring of a saturated structure (without unpaired position) yields a sequence that uniquely folds with respect to base pair maximization. It follows that a competitive/alternative structure may only result from a base pair $(i,j)\notin T$, a position of which is a  node while the other is a leaf. Ensuring that all  nodes and leaves are found at different levels is thus sufficient to guarantee the designability of $T$, i.e. the existence of a solution to this instance of the inverse folding problem.

More generally, we say that a target secondary structure $T$ is *separable* if there exists a coloring $\chi$ such that $\chi$ is separated for $T$. We recall the main results of Halès et al. [17] here.

#### Theorem 1

(Separable $\implies$ Designable [17]) If a tree/secondary structure $T$ is separable, then $T$ is designable.

Moreover, given a separated coloring, an RNA sequence that uniquely folds into $T$, i.e. a solution to the inverse folding problem, can be found in linear time.

#### Remark 1

Note that any design sequence $w$, generated through a separated coloring, avoids any alternative structure featuring GU base pair(s). Indeed, every G and C need to be paired to achieve the number of base pairs featured in the MFE. Meanwhile, the formation of any GU base pair, leaves one C and one A unpaired, resulting in the overall loss of at least one base pair. Structures featuring GU base pairs can thus be safely ignored.

#### Remark 2

Note that an alternative assignment of letters would be C to the unpaired positions, UA for  nodes, AU for  nodes. It has no impact thanks to the symmetry of the base pair compatibility graph as depicted on Fig. 3. In practice, it gives access if desired to double the number of sequences with the ones with the unpaired position at C that could have a slightly different content in terms of G and C even if not studied in this manuscript.

## Separability: intrinsic and computational limits

Despite utilizing separability to explore a design of approximative instances, the work of Halès et al. [17] left open the complexity of searching for a separated coloring, as well as the existence of designable, yet non-separable, structures. An exhaustive search for all structures with up to 12 bases, summarized in Fig. 1, shows that for such small instances, all designable instances are separable.

### Designable structures may not be separable

We first show that non-separable designable instances can be constructed.

**Fig. 6:**
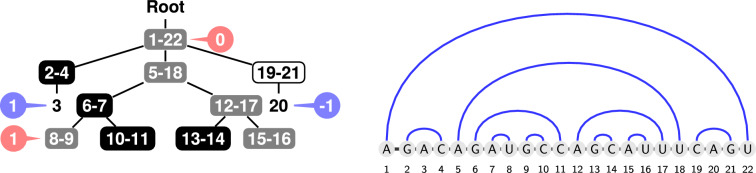
Designability does not imply separability. Left: A target structure that does not admit any separated coloring instance. Note that the coloring $\chi$ shown here puts the  node 8-9 and the leaf 3 both at level 1. Right: Sequence $w$ compatible with the coloring $\chi$, which provably admits $T$ as its single base pair-maximization structure (i.e. $w$ is a design for $T$)

#### Proposition 2

(Designable  Separable) There exists a target structure which: (i) does not admit a separated coloring; and (ii) admits a solution to the inverse folding problem.

#### Proof

We use the tree *T* of Fig. 6 as a counterexample to the notion that separability fully captures designability. First, note that a separated coloring $\chi$ of *T* would be extremely constrained. Node $5-18$ should be  and the nodes $2-4$ and $19-21$ are  and  respectively, or vice-versa due to their respective leaf. Thus, we have two leaves at levels 1 and $-1$. At least, one of the two children of $5-18$, w.l.o.g $6-7$ is  or . One child of $6-7$ is then necessarily , leading to a  child of level $+1$ or $-1$. With two leaves at level $+1$ and $-1$, a direct consequence is that *T* is non-separable.

*Now, we show that* *T* *is designable. We propose the sequence* $w$ *of Fig. **6*. *Using a simple dynamic programming algorithm, it is possible to check that the best folding for *$w$ *is unique and corresponds to the secondary structure encoded as the tree* *T*. *Intuitively, the only competitive alternative base pair is the one corresponding to the overlap of the levels. It consists of joining the *$\textsf {U}$ *from* $8-9$ *with the* $\textsf {A}$ *at position 3. By doing so, note that the base pair *$5-18$ *will be disconnected with no way to pair *$\textsf {A}$ *with another* $\textsf {U}$ *due to the connection between 5 and 7*. $\square$

Notice that, despite not being separated, the coloring shown in Fig. 6 is compatible with a sequence that is a design for its target. This illustrates the fact that, while not being guaranteed to uniquely fold as their intended target, sequences produced from non-separated colorings may still represent solutions for the inverse folding problem.

**Fig. 7:**
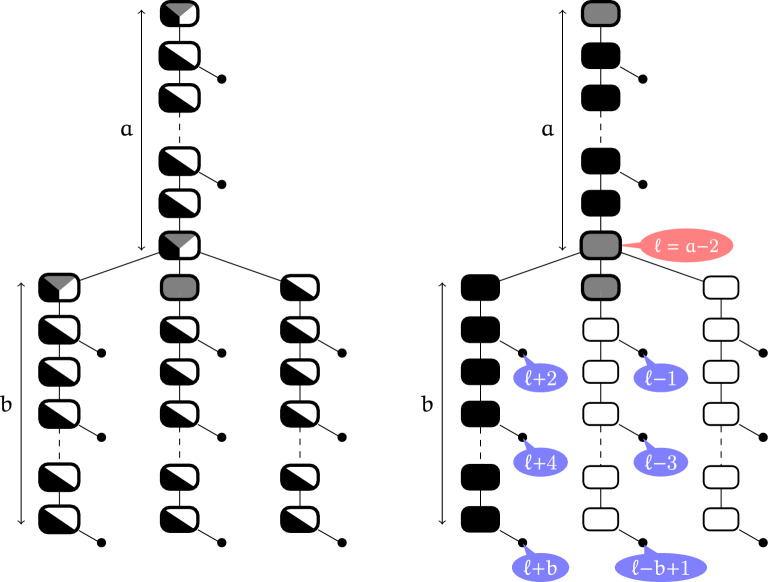
Main gadget used to build non-separable instances with $h_{\min } =2$. Left: Admissible colors for each node (up to branch symmetries). Right: Example coloring and levels of a selection of leaves and  nodes. Note that along with the  node at level $\ell$, there always exists a leaf at level $\ell +m$ or $\ell -m$ for $2\le m\le b$, ruling out modulo separability for small *m*

### Structures with small helices may not be separable

#### Proposition 3

There exist non-separable structures with minimum helix length of 2 (i.e. such that $h_{\min } =2$).

The full proof relies on a counterexample built from the gadget in Fig. 7 and is given in the next paragraph. Intuitively, *T*(*a*, *b*) saturates all levels modulo *b* with leaves, so that none remains available for  nodes. Meanwhile, the presence of multiloops forces proper colorings to use  nodes, so a collision occurs and the gadget is not *m*-separable for any $m\le b$. By assembling 5 copies of *T*(*a*, *b*) with large *b* and increasing values of *a*, we obtain a target that is not separable for any *m*.

We start with the following remark:

#### Proposition 4

If $u_0,\ldots , u_k$ is a path in $T$ and each $u_i$ for even *i* has a leaf attached to it then, for any coloring $\chi$ of the path, we have  and $\chi (u_i)=\chi (u_0)$ for all *i*.

#### Proof

Indeed, by the proper coloring constraint, every node with an attached leaf or with a leaf sibling may not be , so all  for all *i*. Moreover, there can be no direct edge between  and  nodes, so $\chi (u_i)=\chi (u_{i-1})$ for all *i* which gives the desired property by induction. $\square$

With this gadget in hand, we can now build a non-separable structure with $h_{\min }=2$.

#### Proof of proposition 3

We build a non-separable instance *I* without size-1 helix nor $(m_{3\bullet }, m_{5})$ motif. Let $a\ge 2$ and $b\ge 2$ be even numbers. Let *T*(*a*, *b*) be the gadget from Fig 7, containing a length-*a* path from the root to an internal node denoted *t*, and three length-*b* branches attached to *t*. Further attach a leaf to every node at an even distance from the root (except *t* itself). Note that all helices in *T*(*a*, *b*) have length 2. The *level* of a copy of some *T*(*a*, *b*) gadget is the level reached under node *t* of this gadget.

We build the instance *I* as a tree containing 5 copies of the gadget *T*(*a*, *b*), precisely $I=( ((T[10,100], T[20,100])), ((T[30,100], T[40,100])), T[50,100])$.

*First note that for a copy of gadget* *T*(*a*, *b*) *at level *$\ell$ *in any separable coloring, there is a**node at level* $\ell$, *since the node* *t has three children and at least one must be *. *Also, there exists two integers* *u*, *v* *such that, for every* $x\in [ 1,b[$, *there is a leaf at level* $\ell +ux$ *if* *x is odd, and level* $\ell +vx$ if *x* i*s even. Indeed, pick one gray child* *U* of *t*,* and one non-gray child* *V*. *All vertices under* *U form an all-white or all-black branch by Proposition* 4*(we let respectively* $u=-1$ *and* $u=1$*), and vertices at levels* $l+u, l+3u, \ldots , l+bu$ *(or* $l+(b-1)u$*) have a pending leaf. We similarly define* $v=1$ *if* *V* is black and $v=-1$ *if* *V is white, and vertices at levels* $l+2v, l+4v, \ldots , l+bv$ *(or* $l+(b-1)v$*) have a pending leaf. From the above, if there are**nodes at levels* $\ell _1$ *and* $\ell _2$ *with* $\ell -b\le \ell _1<\ell <\ell _2 \le \ell +b$*, then* $\ell _1\ne \ell _2\mod 2$ (since otherwise, one of $\ell _1,\ell _2$ *could be written as* $\ell +ux$ *with even* *x*,* so that level would be a leaf level).*

Aiming at a contradiction, assume that *I* admits a separable coloring. Let $\ell _1\le \ell _2\le \ell _3\le \ell _4\le \ell _5$ be the levels of all five copies of the *T*[*a*, *b*] gadgets of *I*, in ascending order. Then from the length of the branches from the root, we have $\ell _i\in [-50,50]$ and $\ell _i\ne \ell _j$. Then by the remark above applied to the gadget with level $\ell _2$, we have $\ell _1\not \equiv \ell _3\mod 2$, and similarly using gadgets with level $\ell _4$ we have $l_3\not \equiv l_5 \mod 2$ and $l_1\not \equiv l_5\mod 2$, leading to a contradiction (any three integers such as $\ell _1$, $\ell _3$ and $\ell _5$ may not have pairwise distinct parities). $\square$

### Computational hardness of deciding separability

Regarding computational complexity, although looking for a separable coloring is not directly equivalent to finding a design for a structure, we show that this decision problem (formalized below) is also NP-complete.

#### Problem 2

Separability

**Input:** Target tree $T$ (without any occurrence of $m_{3\bullet }$ or $m_{5}$ motif)

**Output:** Coloring $\chi$ of the tree $T$ such that $\chi$ is separated

#### Theorem 5

Separability is NP-complete.

We further show that even when isolated base pairs are forbidden in the input structure (e.g. helices are all of size 2 or more), the separability problem is still NP-hard. Thus, unless $P=NP$, the hope to find a polynomial algorithm for separability holds only when helices are of size 3 or more:

#### Problem 3

2-Helix Separability

**Input:** Target tree $T$ (without any occurrence of $m_{3\bullet }$ or $m_{5}$ motif) whose corresponding target structure contains no isolated base pair ($h_{\min }=2$)

**Output:** Coloring $\chi$ of the tree $T$ such that $\chi$ is separated

#### Theorem 6

*2-helix separability is NP-complete*.

Clearly, Theorem 5 follows from Theorem 6, since the latter relates to a strictly more general problem. We first give an outline of the reduction below, then provide the full proof in the following subsections.

Although proper colorability is a local constraint, separability implies a form of synchronization between different branches of the tree, since a conflict can appear between a leaf and a  node even if they are in remote sections of the tree. However, we do not have a direct way to enforce that a specific level has  nodes or leaves, since there is a lot of freedom in proper coloring constraints, especially in trees with $h_{\min }=2$. The first building block for our reduction is a *blocking* gadget that saturates one parity (either odd or even levels) in some interval with leaves. This is matched with a constant-size *synchronization* gadget where two levels of different parities necessarily have  nodes. So, even if both gadgets are present in different branches, they must be placed at different levels.

Using these two gadgets, we build our reduction from Bin Packing with a tree using one branch per item. Each item has a blocking gadget having the size of the item surrounded by two synchronization gadgets. This enforces that items must be packed in non-overlapping ranges of levels. Additional synchronization gadgets further enforce that series of consecutive items sum up to the target bin size, thus enforcing that items are ordered according to a correct bin packing. However, the synchronization gadget induces some margin of freedom on the specific position of the  levels, so consecutive items may be misaligned by some constant margin. This leads to a formulation of Bin Packing as an interval packing problem, with *blurred* endpoints with some constant margin *L*.

#### Formulation of bin packing as *L*-blurred interval packing

2-helix separability is clearly in NP, since any coloring (certificate) can be checked in linear time. We prove hardness by reduction from bin packing which we formulate as an interval packing problem using unary encoding and *blurred* endpoints.

##### Definition 5

Given a set of pairwise distinct even integers $A = \{a_1, \cdots , a_n\}$, integers *k* and *B* with $kB=\sum _{i=1}^n a_i$ and a constant *L*, an *L-blurred interval packing* of (*A*, *k*, *B*) is a set of integers $u_i,v_i$ for each $1\le i\le n$ and $x_j$ for $0\le j\le k$ such that:

- $-L\le x_j\le kB+L$ for all *j* and $x_0-L\le u_i,v_j \le x_k+L$
- $$|x_{j+1}-x_j|\le B$$
- $$v_i\in [u_i+a_i - L, u_i+a_i+L]$$
- for $i\ne i'$, intervals $[u_i,v_i[$ and $[u_{i'},v_{i'}[$ have an intersection of size at most *L*
- there is no *i*, *j* such that $x_j \in ]u_i+L, v_i-L[$.

Let $A_0=\{\alpha _1,\ldots ,\alpha _n\}, k, B_0$ be an instance of Bin Packing with $kB_0=\sum _{i=1}^n \alpha _i$ and *L* be any constant. Let *M* be the smallest even integer with $M>(n+4)L$. Write $a_i= M\alpha _i$ and $B=MB_0$.

##### Lemma 1

The following are equivalent:

1. $(A_0,k,B_0)$ is a yes-instance of Bin Packing
2. (*A*, *k*, *B*) admits an *L*-blurred interval packing
3. (*A*, *k*, *B*) admits a 0-blurred interval packing

##### Proof

*We show* $$1.\Rightarrow 3. \Rightarrow 2. \Rightarrow 1.$$.

$1.\Rightarrow 3.$ *Set* $x_j = jB$ *for each* $0\le j\le k$.* Let *$(p_1,\ldots ,p_n)$ *be a permutation of [1,* *n*] *such that bin 1 contains elements* $\alpha _{p_1}, \alpha _{p_2},\ldots ,\alpha _{p_m}$ *for some m, then bin 2 contains elements* $\alpha _{p_{m+1}}, \alpha _{p_{m+2}},\ldots ,\alpha _{p_{m'}}$ *for some* $m'$*, etc. Define *$u_i$ *with* $u_{p_1}=0$, $u_{p_{i+1}}=u_{p_i}+a_{p_i}$, *and* $v_i=u_i+a_i$.* Then the first four conditions are trivially verified. For the final condition, for each* *j*, t*he items in the first *$j-1$ *bins have sizes summing to exactly* $(j - 1)B$*, so there is some* *i* *such that* $u_i=x_j$*, and by the fourth condition there is no* $i'$ *with* $u_{i'}+L< x_j < v_{i'}-L$.

$3.\Rightarrow 2.$.* Trivial, all conditions are weaker for L-blurred interval packing than 0-blurred interval packing.*

$2.\Rightarrow 1.$ *We start with the following observation: by the constraints 1. and 2., one can have* $x_j<x_{j-1}$*. However, considering only indices such that* $x_j\ge x_{j-1}$*, the union of intervals* $[x_{j-1},x_j[$ *contains at least* $[x_0,x_k[$*. Let* $I_j$ *be the set of indices i* *such that* $x_{j-1}-L \le u_i < x_j-L$*. Sets* $I_j$ *form a partition of [1, n*] *(it is clear that they are disjoint, and each *$i\in [1,n]$ *is in some *$I_j$ *since otherwise* $u_i<x_0-L$ *or* $u_i\ge x_k-L$ *so* $v_i> u_i+L\ge x_k$*). For each* *j*, *and* $i\in I_j$*, we have *$v_i\le x_j+L$*, so interval* $[u_i,v_i[$ *is included in* $[x_{j-1}-L, x_j+L[$*. Each interval has size between *$a_i-L$ *and* $a_i+L$*, and these intervals overlap on at most* *L* *positions, so* $\sum _{i\in I_j} a_i\le x_j-x_{j-1}+|I_j|L+2L\le B+(n+2)L$*. Since each* $a_i$ *and* *B is a multiple of *$M>(n+2)L$*, we have *$\sum _{i\in I_j} a_i\le B$*, and* $\sum _{i\in I_j} \alpha _i\le B_0$*: sets* $I_j$ *form a solution of *Bin Packing$(A_0,k,B_0)$. $\square$

#### Reduction from interval packing to separability

The reduction is based on two gadgets called *blocking* and *synchronization* gadgets. The first gives a long chain of nodes with a leaf attached to every other node; we show that this enforces a long interval of levels with leaves at all odd or even level. The second has a fixed size, and is incompatible with a blocking gadget since any separated coloring has both an odd and an even  level. Both gadgets are defined in the following paragraphs, as well as their main properties. These properties are formulated in terms of *synchronized* and *blocked* levels, defined now.

We set $H=12$ (chosen as the height of the synchronization gadget defined below), and use a blur value $L=3H+2$.

A level *u* is *H-synchronized* if there are two  levels with different parity in $[u-H,u+H]$.

A level *u* is *H-blocked* if either all odd or all even levels in $[u-H,u+H]$ are leaf levels.

##### Observation 1

In any separated coloring, no level can be both *H*-synchronized and *H*-blocked.

***Blocking gadget***

A *blocking gadget* of size *q* in a tree is a chain of *q* node $s_1,\ldots , s_q$ with a leaf attached to $s_i$ for each odd *i*.

##### Proposition 7

In any proper coloring of a size-*q* blocking gadget, all nodes have the same  or  color. Furthermore, let $\ell _1,\ell _2$, be the levels above the root and below the last node of the chain, such that $\ell _1\le \ell _2$. Then $\ell _2=\ell _1+q$ and all levels in interval $[\ell _1+H,\ell _2-H]$ are *H*-blocked.

##### Proof

By the proper coloring condition, no parent or sibling of a leaf can be , and 2 adjacent non- nodes must have the same color, so all nodes $s_i$ are given the same non- color. Furthermore, if the gadget is colored , then $\ell _1$ is the level above the root, and $\ell _2=\ell _1+q$. Plus, there are leaves at levels $\ell _1 + 2i+1$ for all *i* such that $1\le 2i+1\le q$, so all levels *j* with $\ell _1+H\le j\le \ell _2-H$ are *H*-blocked. Similarly if the gadget is colored , then $\ell _2$ is the level of the root, $\ell _1=\ell _2-q$, and again levels $\ell _1+H\le j\le \ell _2-H$ are *H*-blocked. $\square$

***Synchronization gadget***

**Fig. 8:**
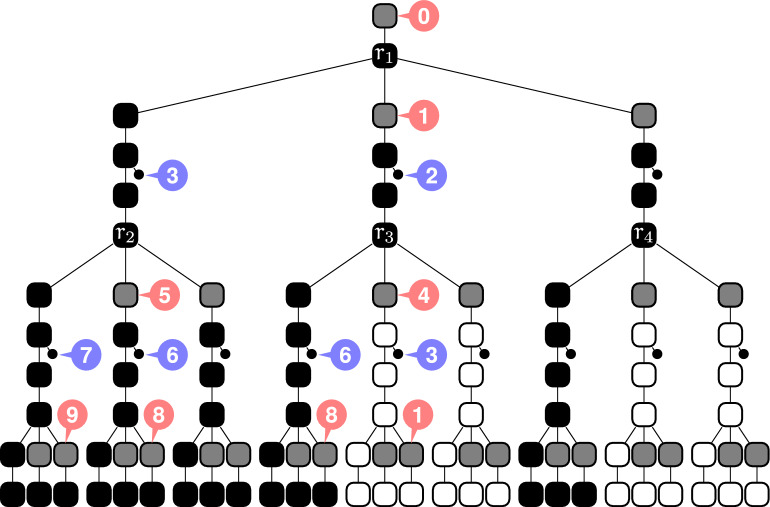
The synchronization gadget with a proper coloring of its nodes using leaf levels $\{2,3,6,7\}$ and  levels $\{0,1,4,5,8,9\}$. By Lemma 2, any proper coloring has two  levels with distinct parity so the root level is *H*-blocked

The main gadget for our reduction is a fixed-sized tree for which any separated coloring uses two  levels with distinct parity (see Lemma 2 below).

##### Lemma 2

The *synchronization gadget* shown in Fig. 8 admits a separated coloring with  root and using only leaf and  levels in $[r+0,r+9]$ (where *r* is the level of its root). Moreover, for any separated coloring the level of the root is *H*-synchronized.

##### Proof

Let *r* be the level of the root. The coloring with gray levels in $[r+0,r+9]$ is given in Fig. 8.

*For the main part of the proposition, assume that the gadget admits a coloring *$\chi$ *such that all**nodes have the same level parity. Since all distances to the root are at most* *H*, *we need to ensure that there are two**nodes with levels at different parity anywhere in the gadget*.

*Suppose first that some node* $r_i$ ($i\in \{1,2,3,4\}$*) of the gadget is colored **, then among the 3 chains below* $r_i$*, one starts with a**node, one starts with a**node, and the last with a**node. We denote the nodes of the chain starting by*$c_1$, $c_2$, $c_3$ *and* $c_4$ *with* $c_1$ a *node and the chain starting by*$b_1$, $b_2$, $b_3$, $b_4$.* Note that *$b_1$ *and* $b_2$ *are*. $c_2$ *can only be**or**as it has a leaf child (w.l.o.g. we assume it is**).* $c_3$ *should also be **due to the leaf of* $c_2$ *and to the proper condition.* $c_4$ *should also be**to avoid conflict with the leaf child of *$b_2$*. Thus,* $c_4$ *has necessarily a**child and it has a parity different from the **node* $c_1$ *as there are 3**nodes between them.*

*Suppose now that each *$r_i$, $i\in \{1,2,3,4\}$ *is non-**. Consider* $r_1$*, we again denote the four vertices starting on chain starting on a **node* $c_1$*,* $c_2$*, *$c_3$ *and* $c_4$*. Then* $c_1$ *is**, *$c_2$ *and* $c_3$ *have the same non-**color (again because of the leaf attached to* $c_2$*), and* $c_4=r_i$ *for some* $i\in \{2,3,4\}$ *also has the same non-*color. Let $c_1'$ *be a**children of* $c_4$*: the level difference between* $c_1$ *and* $c_1'$ *is 3, so they have different parity, which concludes the proof.*

$$\square$$

***Object gadget***

An *object gadget* of size *a* (with *a* even and $a\ge H$) is a chain of $a+1$ nodes, with two synchronization gadgets attached respectively to the first and last nodes in the chain, and a leaf attached to the *i*th node for each even $i>H$.

##### Proposition 8

If an object gadget of size *a* appears in a tree with a separated coloring $\chi$, there exist levels $u\le v$ such that:

- levels *u* and *v* are *H*-synchronized
- $$a-L \le v-u \le a+L$$ (recall that $$L\ge 3H+2$$)
- all levels in $[u+L, v-L]$ are *H*-blocked.

##### Proof

We define *u* and *v* as the levels of the roots of both synchronization gadgets (with $u\le v$). Both *u* and *v* are *H*-synchronized by Lemma 2. Write $b_1,b_2$ respectively for the $(H+1)$th and *a*-th node of the object gadget. The chain from $b_1$ to $b_2$ form a blocking gadget of size $a-H$. Let $u',v'$ be the levels above $b_1$ and below $b_2$ respectively, with $u'\le v'$. By the distance in the tree, $|u'-u|\le H+1$ and $|v'-v|\le H+1$. Moreover, by Proposition 7, $|v'-u'|=a-H$ (so $a-3H-2\le |v-u|\le a+3H+2$) and all levels in $[u'+H,v'-H]$ (that contains $[u+L,v-L]$) are *L*-blocked. $\square$

**Fig. 9:**
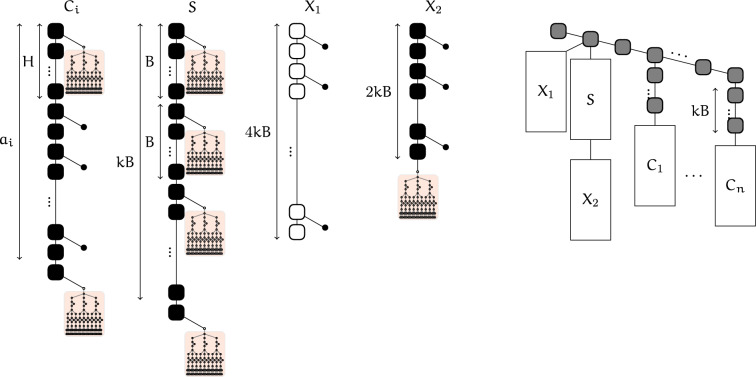
Left: details of the four main parts of the reduction, i.e. an object gadget $C_i$ of size $a_i$, the chain *S*, and blocking gadgets $X_1$ and $X_2$). Right: general layout of the tree built in the reduction

Given an instance *A*, *k*, *B* of *L*-Blur Interval Packing, we build a tree *T* as follows (Fig. 9):

- We start with a chain *P* of length 2*n*, with vertices denoted $q_0,p_0,q_1,p_1,\ldots ,q_n,p_n$. (In order to avoid isolated base pairs, we only attach subtrees to nodes $p_i$, not $q_i$).
- For each $i \ge 1$ we attach a chain (denoted $P_i$) of *kB* nodes to $p_i$ followed by an object gadget $C_i$ of size $a_i$.
- We attach a blocking gadget $X_1$ of size 4*kB* to $p_0$.
- We attach a long chain to $p_0$ composed successively of:
  - a chain *S* of $kB+2$ nodes with a synchronization gadget attached to the $(iB +2)$th node for each $0\le i \le k$
  - a subtree $X_2$ composed of a blocking gadget of size 2*kB* with a synchronization gadget attached to the last node.

#### Correctness proof

We now complete the correctness proof of the reduction with the following Lemma.

##### Lemma 3

We have the following two implications

$$\begin{aligned}&(A,k,B) \text { admits a 0-blurred interval packing} \Rightarrow T \text { is separable} \\&T \text { is separable} \Rightarrow (A,k,B) \text { admits an }L\text { -blurred interval packing} \end{aligned}$$

The proof is given in the following two sections. This lemma completes the proof of Theorem 5, since together with Lemma 1 we have that $(A_0,k,B_0)$ admits a Bin Packing if and only if *T* is separable. Moreover, using the strong NP-hardness of Bin Packing, we can assume that all integers $\alpha _i$ are bounded by a polynomial in $|A_0|$ (corresponding to a unary encoding), so *T* can be built in polynomial time from $(A_0,k,B_0)$. Finally, it can easily be checked that *T* does not have isolated base pairs (however, *T* does contain isolated stacks, so $h_{\min }=2$).

***From 0-blurred interval packing to separated coloring***

**Fig. 10:**
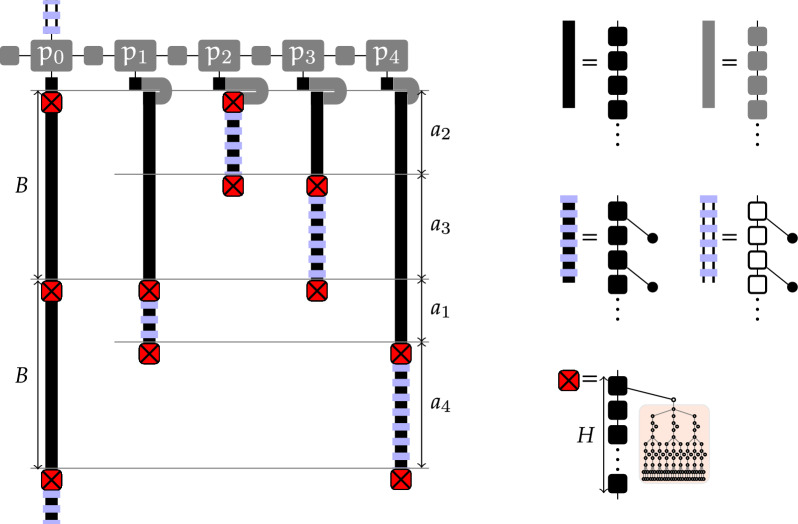
Example of the reduction with $n=4$ items with sizes $\{a_1,a_2,a_3,a_4\}$ to be sorted into $k=2$ size-*B* bins. Each item is mapped into a branch $P_i$ followed by an object gadget $C_i$, containing 2 synchronization gadgets (shown as red nodes with crosses) separated by the size of the item. Leaves in object gadget enforce that any two gadgets may overlap only if the synchronization gadgets are aligned (within a margin of *L* levels). The bins are implemented using the chain *S*, with synchronization gadgets at every *B*th position, enforcing that series of consecutive items are packed into size-*B* bins. Finally, blocking gadgets $X_1$ and $X_2$ may not overlap with any synchronization gadget, and enforce that all object gadgets as well as the chain *S* are packed together in a size-*kB* range of levels

We consider a 0-blurred interval packing assigning integers $u_i,v_i$ to each item $a_i$ (and $x_j=jB$) as defined in Fig. 10. In words, chain *P* is colored . Each chain $P_i$ ($i\ge 1$) starts with a  node, ends with $u_i$ nodes, and all remaining nodes are . All synchronization gadgets are colored as in Fig. 8. The chain $X_1$ is , and all other nodes are colored .

We show that this coloring is separated. Note that  nodes either have level 0 or 1, or are part of a synchronization gadget. Let $X=\{u_i\mid 1\le i\le n\}\cup \{kB\}$, all synchronization gadgets have level $x\in X$, so  nodes have levels in $\{0,1\}\cup \bigcup _{x\in X} [x,x+9]$. Since synchronization gadgets are separated (locally), it remains to verify that no leaf in the rest of the tree has a level in this set. Indeed, leaves in $C_i$ have levels between $u_i+L$ and $v_i-2$, while $X_1$ and $X_2$ have leaf levels $<0$ or $>kB$.

***From separated coloring to L-blurred interval packing***

Suppose now that *T* admits a separated coloring $\chi$. Assume that the level below node $p_0$ is 0 (otherwise, apply an offset to all level values below). Consider first blocking gadget $X_1$. By Proposition 7, it is either colored  or . Without loss of generality, assume it is colored . Then since it has size 4*kB*, all levels in $[-4kB+H, -H]$ are *H*-blocked. In particular, since there is no path of length $\ge 4kB-H$ in the rest of the tree, all synchronization gadgets must have a level $\ge -H$.

Consider now the blocking gadget $X_2$, let *N* be the level of its first node. We have $|N|\le kB+1$. If $X_2$ is colored , then the synchronization gadget below it would be at level $N-2kB\in [-4kB+H, -H]$, which is not possible (this level is blocked), so $X_2$ is colored , and all levels in $[N+H, N+2kB-H]$ are *H*-blocked. Since all synchronization gadgets in the *S* or $C_i$ chains are at distance at most $2n+kB+\max a_i +2$ from the root, and this distance is upper bounded by $N+2kB-H$ (with the reasonable assumption that *B* is large enough with respect to *n*, precisely $kB\ge 2n+\max a_i +2$), they all have level at most $N+H-1\le kB+H\le kB+L$.

Write $x_j$ for the level of the *j*th synchronization gadget in *S*: $x_j$ is a synchronized level. By the size of paths between successive gadgets, $|x_{j+1}-x_j|\le B$, and by the remark above, $-L\le x_j\le kB+L$.

For each object gadget $C_i$, by Proposition 8, there exist *H*-synchronized levels $u_i,v_i$ such that $v_i\in [u_i-L,u_i+L]$, and such that all levels in $[u_i+L,v_i-L]$ are *H*-blocked. Overall, we have integers $x_i, u_i,v_i$ satisfying the conditions for an *L*-blurred interval packing.

## Modulo separability as a parameterized tractable alternative

**Fig. 11:**
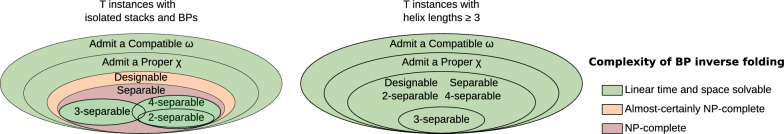
Instances of Inverse-Folding_BP_ . For unconstrained instances (Left), Inverse-Folding_BP_ is likely NP-hard, as suggested by the hardness of a constrained version [3]. Finding a design for a separable target is also NP-hard but, for any fixed modular level *m*, *m*-separable targets can be designed in $\Theta (n)$ time. This suggests an algorithm, FPT on *m*, for all separable structures. When $h_{\min } \ge 3$ (Right), Theorem 12 applies and the hierarchy collapses: any instance becomes 2-separable ($\implies$ separable and designable) and Inverse-Folding_BP_ can be solved in $\Theta (n)$ time

Then, we introduce a stratified version of separability, called modulo *m*-separability, or *m*-separability in short, which prescribes different modular values for the levels of  and leave nodes. Figure 11 describes the relative positioning of classes of instances and associated complexity results.

### Definition 6

((Modulo) *m*-separability) Let *m* be an integer. A coloring $\chi$ is *m-separated* (or *separated with modulus m*) for a target secondary structure $T$, if and only if $\chi$ is proper and
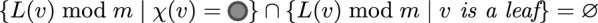


using for negative levels $l<0$ the classic $l\bmod m:= \left( l+\lceil -x/m\rceil \times m\right) \mod m$.

A structure is *m-separable* if it admits an *m*-separated coloring.

Clearly, modulo separability implies classic separability: if a coloring $\chi$ is *m*-separated for a target structure $T$, then $\chi$ is separated for $T$. Conversely, if a target structure admits a separated coloring, assigning levels in $[-a,b]$ to  and leaf nodes, then the same coloring is provably $m'$-separated for $m':=(b+a+1)$ (since, for $l,l' \in [-a,b]$, $l\ne l'$ implies that $l\bmod m' \ne l'\bmod m'$). Note that, since there are at most *n*/2 base pairs/internal nodes in a target tree, then $0\le a,b\le n/2$, and we have $m'\le n$.

The concept of *m*-separability thus provides an angle to address the generation of separated colorings, so we introduce below the associated formalized algorithmic problem.

### Problem 4

Modulo Separability

**Input:** A tree $T$ (with no $m_{3\bullet }$ or $m_{5}$ motif), a modulus $m\in \mathbb {N}$

**Output:** A coloring $\chi$ of $T$ that is *m*-separated, or $\perp$ if no such coloring exists.

As noted above, the problem specializes in the Separability problem when $m=n$, implying that Modulo Separability remains NP-complete. However, it can be efficiently solved for moderate values of *m*, as shown below. Practically, one may focus on small values of *m* since 99% of instances without isolated base pairs are separable with modulus $m\le 6$ (*cf* Table 13).

### Fixed parameter tractable algorithm for modulo-separability

We now show that, for any fixed modulus *m*, Modulo Separability can be solved in linear time. In particular, the problem is Fixed Parameter Tractable (FPT) for the parameter *m*.

Towards that goal, we consider a constrained version of Modulo Separability, where the modular values of levels are prescribed. Formally, we enforce that leaves only occur at modular levels in $\xi _L\subseteq [ 0,m[$, and  nodes only occur at levels $[ 0,m[ \setminus \xi _L$. In this constrained version of Modulo Separability, the existence of a valid solution can be solved in linear time using dynamic programming.

Namely, let us denote by $\text {d}^{\xi _{L}}_{v\rightarrow c, \ell }$ the existence of a valid assignment (*i.e. solution*) for a subtree of $T$ rooted at internal node *v*, with *v* occurring at level $\ell$, and being assigned a prior color *c*. Provably, $\text {d}^{\xi _{L}}_{v\rightarrow c, \ell }$ can be computed recursively by progressing along the tree, keeping track of the current level and checking that leaves and  end up being assigned at modular levels $\xi _L$ and $[ 0,m[ \setminus \xi _L$ respectively. This leads to the following formula:


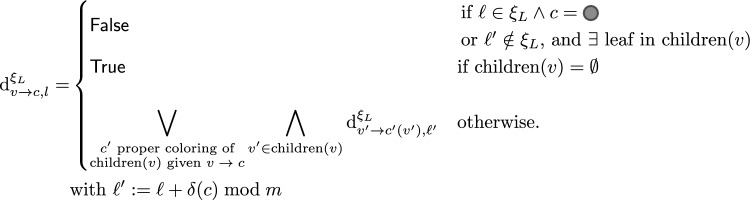


where $\delta$ denotes the level increment induced by a color *c*, defined as ,  and . Moreover, in the outermost loop, the color assignment explored for children is meant to be locally proper: the colors $c(v')$ of the children, in conjunction with the color *c* of *v* must obey the conditions of Definition 2. Note that, in the absence of $m_{3\bullet }$ and $m_{5}$, the number of (proper) assignments is bounded by a constant, so this conjunctive loop does not impact the complexity. The existence of a $\xi _L$ coloring for the full tree is then $\text {Separable}_{\xi _L}:= \text {d}^{\xi _{L}}_{\textsf {Root}\rightarrow \varnothing , 0}.$

The decision version of the problem can thus be solved in $\Theta (m.n)$ time. Indeed, the number of left-hand side terms scales in $\Theta (m.n)$, the number of proper coloring for children is bounded by a constant (since avoiding $m_{3\bullet }$ and $m_{5}\implies |\text {child}(v)|<5$), and the total number of executions of the conjunctive loops is in overall $\Theta (n)$. A backtracking procedure could also be defined to reconstruct a solution coloring in $\Theta (n)$ if such a solution exists ($\text {Separable}_{\xi _L}=\textsf {True}$) or return $\perp$ otherwise ($\text {Separable}_{\xi _L}=\textsf {False}$).

An algorithm for modulo separability can then be obtained by explicitly considering all the possible subsets of admissible modular levels for leaves:

- If $T$ contains $m_{3\bullet }$ or $m_{5}$, return $\perp$
- For each $$\xi _L\subseteq [ 0,m[$$:
  - If $\#\text {Designs}_{\xi _L}>0$, then backtrack to produce $\xi _L$-separated design
- Return $$\perp$$

The algorithm is correct: for any *m*-separated coloring $\chi$, there exists at least one $\xi _L \subseteq [ 0,m[$ corresponding to the leaves of $\chi$ solution and any solution to the *m*-separated property implies a partition of the leaves and  nodes into disjoint levels $\xi _L$ and  respectively. An *m*-separated coloring is thus always found by invoking the DP algorithm over the $2^{m}$ subsets $\xi _L\in [ 0,m[$. The overall complexity of the algorithm is in $\Theta (n.m.2^m)$ time and $\Theta (m.n)$ memory, and we conclude with the parameterized complexity of the problem with respect to *m*.

#### Theorem 9

Modulo Separability is Fixed Parameter Tractable for the modulus parameter *m*

### Random generation of *m*-separated sequences

We then turn to the uniform random generation of *m*-separated sequences, defined below: First, for convenience, given a sequence $\omega$ compatible with a structure *T*, we define $\chi _w$, the associated coloring of *T* obtained by replacing base pairs with suitable color (i.e. ,  and )

Then we define a proper (resp. m-separated) sequence $\omega$ as a sequence that would be obtained from a coloring $\chi _w$ itself proper (resp. m-separated). As will be shown in Sect. Any *m*-separated sequence is a valid design any such sequence represents a design for its respective target structure $T$. More formally, a sequence $w$ for $T$ is *proper (resp. m-separated)* if:

1. The coloring $\chi _w$ is proper (resp. *m*-separated);
2. Only $\textsf {A}$ nucleotides are found at unpaired positions and $w$ is compatible with $T$;
3. The contents of two  base pairs present within a multiloop or both direct children of the root must; (i) differ if they are siblings; and (ii) coincide if one is the parent of the other, noting that at most two  nodes may coexist within a multiloop.

Note that a *m*-separated sequence is also a proper sequence (A $\chi _w$ *m*-separated is proper by definition since 2. and 3. are inherited without modifications).

#### Problem 5

Uniform Modulo Separated Generation

**Input:** Target tree $T$ (with no $m_{3\bullet }$ or $m_{5}$ motif)

**Output:** RNA sequence $w$, associated with *m*-separated coloring $\chi _w$, such that

$$\mathbb {P}(w\in \Sigma ^n m\text {-separated}) = \frac{1}{|\{w' \in \Sigma^n \mid w' \text{ is } m\text {-separated} \}|}$$

#### Linear-time uniform sampling for fixed modular assignments

Once again, we approach this problem by first solving a more constrained version where the modular levels of leaves are explicitly given as a set $\xi _L$, denoted as *modular assignment* in the following. Then, in the spirit of Reinharz et al. [12], we adapt the above recurrence, through a simple algebra change, to count the number $\text {p}^{\xi _{L}}_{v\rightarrow \mu , l}$ of RNA sequences, associated with what we call a $\xi _L$ *separated coloring*, that is to say an *m*-separated coloring such that all leaf levels are $\xi _L$. (for a subtree of $T$ rooted at *v*, with *v* occurring at level *l*, and being assigned a nucleotide assignment $\mu$).

$$\begin{aligned} \text {p}^{\xi _{L}}_{v\rightarrow \mu , \ell } =&{\left\{ \begin{array}{ll} 0& \text {if } \ell \in \xi _L\text { and }\mu \in \{(A, U), (U, A)\}\\ 0& \text {if } \ell ' \notin \xi _L\text { and }v \text { has a leaf attached}\\ 1& \text {if children }(v)=\varnothing \\ \displaystyle \sum _{\begin{array}{c} \mu ' \text { proper assignment}\\ \text {children}(v) \rightarrow \Sigma ^2\cup \{\emptyset \} \end{array}} \prod _{\begin{array}{c} v' \in \text {children}(v) \end{array}} \text {p}^{\xi _{L}}_{v'\rightarrow \mu '(v'), \ell '}&\text {otherwise.} \end{array}\right. }\\&\text { with }\ell ':= \ell +\delta (\mu ) \bmod m \end{aligned}$$

where $\mu '$ is a function assigning nucleotides to the children of *v*, consistent with a proper coloring and additionally respecting natural constraints on the content ($(\textsf {A},\textsf {U})$ or $(\textsf {U},\textsf {A})$) of pairs of  nodes (same for both if one parent of other, different content if siblings). Once again, the colorless Root node needs to be distinguished, and the overall number of designs is given by $\#\text {Designs}_{\xi _L}:= \text {p}^{\xi _{L}}_{\textsf {Root}\rightarrow \varnothing , 0}.$

We next propose a backtrack procedure $\textsf {backtrack}^{\xi _L}_{v \rightarrow \mu , \ell }$ with exactly the same parameters as $\text {p}^{\xi _{L}}_{v\rightarrow \mu , \ell }$ and that processes exactly the same cases and then produces a uniform random RNA sequence that corresponds to a *m*-separated coloring for a fixed set $\xi _L$. In that case, by abuse of language, we say that the sequence is $\xi _L$-*separated*. More precisely, $\textsf {backtrack}^{\xi _L}_{v \rightarrow \mu (v), \ell }$ produces a random sequence, associated with a $\xi _L$ separated coloring, for the subtree anchored in *v*, reached at height $\ell$, where the root is assigned a pair of bases $\mu \in \Sigma ^2$. It first picks a random proper assignment $\mu '$ for the children, weighted by the corresponding number of solutions (namely, $$\prod _{\begin{array}{c} v' \in \text {children}(v) \end{array}} \text {p}^{\xi _{L}}_{v'\rightarrow \mu '(v'), \ell '}$$, with $\ell ':=\ell +\delta (\mu )\mod m$). The resulting sequence is then

$$\prod _{\substack{v' \in \text {children}(v) \\\text{or leaves}(v)}}{\left\{ \begin{array}{ll} \textsf {A} \\ \text { If }v'\text { is a leaf}\\ b.(\textsf {backtrack}^{\xi _L}_{v' \rightarrow \mu '(v'), \ell '}).b' \\ \text { otherwise, with } \mu '(v')=b.b'\\ \end{array}\right. }$$

The resulting algorithm, consisting of precomputing all $\text {p}^{\xi _{L}}_{v\rightarrow \mu , \ell }$, followed by a sequence of *k* backtracks, provably returns *k* random, uniformly-distributed and independent designs that are $\xi _L$ separated in time $\Theta (n.m + k.n)$.

#### Any *m*-separated sequence is a valid design

In this section we aim at proving Theorem 10, establishing that all sampled sequences are designs. Additional arguments are needed beyond the original proof of Hales et al. [17] that only give existential results: under properness and separation conditions, the structures admit at least one design. We need here a different kind of result showing that all *m*-separated sequences are designs. Our argument is an adaptation of Theorem 8 from Hales et al. [17]: we first consider the case of *saturated* structures, then, this case is used to prove the more general case, that relies on separability.

##### Definition 7

(Saturated structure, saturable sequence (restatement from Hales et al. [17])) A structure is said to be *saturated* if it contains no unpaired position. A sequence is said to be *saturable* if it is compatible with such a structure.

##### Definition 8

(Atomic saturable design (restatement from Hales et al. [17])) An *atomic saturable* design is a design with no saturable proper prefix.

##### Lemma 4

Proper sequences $w$ over $T$ such that $\chi _w$ is saturated are designs.

##### Proof

We first show that internal nodes give rise to atomic saturable designs by induction over the structure *T*: Assume the current node is a base pair at index positions (*u*, *v*) with children at index positions $(u^i,v^i)_{1 \le i \le t}$. We denote $w^i$, the sub-sequence of $w$ between positions $u^i$ and $v^i$ and $T^i$, the sub-structure made of the base pairs encapsulated between index $u^i$ and $v^i$. The $(T^i)_{1 \le i \le t}$ are saturated (by definition) and their corresponding sub-coloring $(\chi _w^i)$ restricted to $T^i$ are proper (by definition). Thus, with the induction hypothesis, $(w^i)_{1 \le i \le t}$ are atomic saturable designs. Then, we want to use Claim 4.3 from Hales et al. [17] to say that if $\forall 1 \le i \le t: w_1^i \ne w_v$ and $\forall 1 \le i < j \le t: w_1^i \ne w_1^j$ (*) then the concatenation $w= w_u \cdot w^1 \cdot \ldots \cdot w^t \cdot w_v$ is an atomic saturable design. We need to check the condition (*) of the claim: it is a consequence of the sequence $\omega$ being proper (in particular 3. of the definition): no base pair can be assigned the opposite nucleotides of its parent and no two base pairs of a multiloop may be assigned the same nucleotides. We need a specific case for the root. We use Claim 4.2 from Hales et al. [17] saying that if $\forall 1 \le i < j \le t: w_1^i \ne w_1^j$ and if the direct children of the root all generate atomic saturable designs, then their concatenation is a design of the concatenation of the associated structures. Children are indeed atomic saturable designs by induction and again the condition of the claim is a consequence of $w$ being proper (i.e. no two base pairs at the root can be assigned the same nucleotide pair). $\square$

Now that we have managed the saturated case, we can use it for the following result on separability, (for the sake of clarity, let us say that $T$ is a set of base pairs and not a tree):

##### Lemma 5

Let $w$ over $T$ be *m*-separated, and $T'$, a set of base pairs compatible with $w$ then:

- $$\vert T' \vert \le \vert T\vert$$.
- If $\vert T' \vert = \vert T\vert$, then the unpaired nucleotide positions in $\omega$ are the same both for $T$ and $T'$.

##### Proof

We show first $\vert T' \vert \le \vert T\vert$. Since $w$ is *m*-separated (thus proper), $\chi _w$ is proper, then all positions except maybe for some *A*s are paired in $w$: the leaves correspond to unpaired positions, and those are mapped to *A*s by definition. Thus $T'$ may not form more base pairs than $\vert T\vert$ because all *G*s, *C*s and *U*s are paired in $\omega$ with respect to $T$’s base pairs.

*We now prove the second point. To have* $\vert T' \vert = \vert T\vert$*, then *$T'$ *should pair all G*, *C* *and* *U*s in $w$*. By contradiction, suppose there is some positions* (*i*, *j*) *such as* $w_i = A$ *is unpaired in* $T$ *and is paired in* $T'$ *with* $w_j = U$. *Position* *i corresponds to a leaf and* *j* *is involved in a **base pair with respect to the coloring* $\chi _w$ *of* $T$*, thus positions* *i* and *j have distinct levels (with* $\chi _w$ *m-separated). By Claim 8.1 from Hales et al.* [17],* we have that some G* *or* *C (that in fact are positions between* *i* *and* *j*) *are unpaired in* $T'$*. It is in contradiction with* $\vert T' \vert = \vert T\vert$*. Moreover the unpaired nucleotide positions in* $\omega$ *are the same both for *$T$ *and* $T'$. $\square$

We can conclude finally with the theorem:

##### Theorem 10

*m*-separated sequences for $T$ are designs for $T$.

##### Proof

Suppose that there exists $T'$ compatible with $w$ such that $\vert T' \vert \ge \vert T\vert$. By Lemma 5 we have that $\vert T' \vert = \vert T\vert$ and both $T'$ and $T$ have the same unpaired positions over $w$. Thus we can project $w$ onto the set of paired positions, let us denote by $\tilde{w}$ the projection. Note that in this context *T* and $T'$ are saturated over $\tilde{w}$. Similarly, $\tilde{w}$ inherits the proper property from $w$ being *m*-separable and in particular proper. Thus, with Lemma 4 for saturated structures, $\tilde{w}$ is a design for both $T$ and $T'$ on unpaired positions, which implies $T= T'$, which concludes the proof. $\square$

#### Integrating over all modular assignments and correcting for uniformity through rejection

Clearly, a random generation algorithm could be obtained by generating a random modular assignment $\xi _L$ uniformly, and then use the above algorithm to produce a design. However, if naively implemented, such a strategy would suffer from multiple shortcomings:

1. It may not always produce a design, even when such a design exists. Indeed, a naive choice of $\xi _L$ may lead to zero $\xi _L$ separated design;
2. The overall generation scheme would not be uniform over the set of *m*-separated sequences (at a fixed *m*). Indeed, the emission probability of a *m*-separated sequence $w$ that is only compatible with a single assignment $\xi _L$ (i.e. populating all levels in $\xi _L$), is then strictly inversely proportional (for a fixed *m*) to the number of designs compatible with $\xi _L$. Such a probability will thus typically differ across modular assignments, inducing a bias. Even if corrected by a suitable correction upon choosing $\xi _L$, this scheme will favor sequences that are compatible with multiple modular assignments, thus motivating further countermeasures.

To correct those issues, and leverage the uniform generation for a fixed $\xi _L$ into a uniform generation of *m*-separated designs, we implement a classic rejection strategy (see [20], pp 77) for a general exposition). It starts by generating some $\xi _L$ according to a suitable distribution, and then uses a suitable rejection to correct the emission probabilities of sequences compatible with several $\xi _L$.

##### Theorem 11

Uniform modulo separated generation can be performed in $\Theta (n.m.2^m)$ average-case complexity, i.e. Fixed Parameter Tractable on the modulus parameter *m*.

We consider a rejection-based approach, which starts by precomputing all $\#\text {Designs}_{\xi _L}$ in time $\Theta (n.m.2^m)$ (see "Random generation of m-separated sequences" section), and accumulates them into $\mathcal {Z}_m:=\sum _{\xi '_L\subseteq [ 0,m[ }\#\text {Designs}_{\xi '_L}$. It then iterates the following steps until a suitable sequence is returned:

1. Choose some $\xi _L\subset [ 0,m[$ with probability $\mathbb {P}(\xi _L) = \#\text {Designs}_{\xi _L}/\mathcal {Z}_m$
2. Generate a ${\xi _L}$ separated sequence $w$
3. Compute the number $\Xi _w$ of $\xi '_L\subset [ 0,m[$ such that $w$ is $\xi '_L$ separated
4. With probability $1/\Xi _w$, accept/return $w$; Reject/restart from **1.** otherwise.

Due to the full reset on each rejection, the emission probability $p_{w}$ of any suitable $w$ does not depend on the prior sequence of rejections (folklore, proven in ([20], pp 77)), and we have:

$$\begin{aligned} p_{w}&\propto \sum _{\begin{array}{c} \xi _L \text { such that }w\\ \text {is }\xi _L\text { separated} \end{array}} \mathbb {P}(\xi _L)\times \mathbb {P}(w\mid \xi _L)\times \frac{1}{\Xi _L} \\&= \sum _{\begin{array}{c} \xi _L \text {such that } w\\ \text { is }\xi _L\text { separated} \end{array}}\frac{\#\text {Designs}_{\xi _L}}{\mathcal {Z}_m} \times \frac{1}{\#\text {Designs}_{\xi _L}} \times \frac{1}{\Xi _w}. \end{aligned}$$

Some terms directly cancel out and, by definition, we have

$$\sum _{\begin{array}{c} \xi _L \text {such that } w\\ \text { is }\xi _w\text { separated} \end{array}} 1 = \Xi _w.$$

It follows that $p_w\propto 1/\mathcal {Z}_m$, a term that no longer depends on $w$, from which we conclude that the overall generation is uniform.

Complexity-wise, a prior accumulation of the $2^m$ terms $\#\text {Designs}_{\xi _L}$, each smaller than $4^m$, into a suitable data structure (see Lorenz and Ponty [21] for details) enables a random choice of $\xi _L$ (Step 1.) in $\Theta (n.m)$. Once $\xi _L$ is chosen, the above DP algorithm uniformly generates $w$ in time $\Theta (m.n)$ (Step 2). The computation of $\Xi _w$ (Step 3) is trivial and consists in identifying, in time $\Theta (n+m)$, the subset $\Phi _w\subseteq [ 0,m[$ of modular levels that are populated by neither leaves nor  nodes in $\chi _w$. Indeed, those levels represent the only degrees of freedom available while choosing a compatible $\xi _L$, the others modular values being forced to either  or leaves. Since such modular values can be independently chosen to be in or out of $\xi _L$, then we have $\Xi _w= 2^{|\Phi _w|}$. Clearly, we have $\Xi _w\le 2^m$, so the expectation of the number of (independent) rejections admits an upper bound in $2^m$, and the overall average-case complexity is in $\Theta (n.m.2^m)$.

## Structures without isolated stacks and base pairs are 2-separable

Although separability does not give a full characterization of designability in general (cf Prop. 2 and Prop. 3), we obtain a much stronger result for structures without small helices, as hinted by the fact that all counter-examples and hardness gadgets heavily use isolated base pairs or isolated stacks in their construction. Indeed, we show that a 2-separated coloring can be constructed for *all* structures without forbidden motifs $(m_{3\bullet },m_{5})$ and $h_{\min } \ge 3$, so indeed all such structures are designable. Since avoiding $(m_{3\bullet },m_{5})$ is a necessary condition for designability, we obtain the stronger characterization stated in Corollary 2.

### Lemma 6

Let $\overline{\xi _L}$ denote the prescribed modular level for  nodes. Consider an helix *H* consisting of 3 BPs or more ($h_{\min }\ge 3$), whose first BP is assigned some color .

Then for each modular level $l\in [0,1]$ for the first BP of *H* and targeted exit modular level $l'\in [0,1]$, there exists a coloring for the rest of *H* such that:

- The modular level of the upcoming nodes, i.e. those immediately following *H*, is $l'$;
- Base pairs can only be -colored at modular level $\overline{\xi _L}$.

**Fig. 12:**
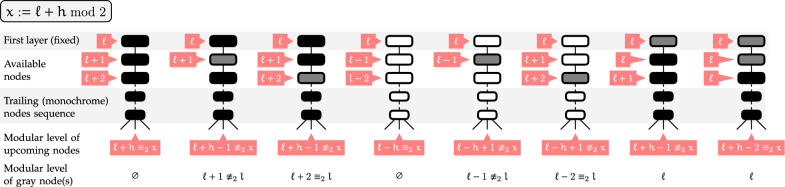
Alternative colorings for helices consisting of 3+ base pairs ($h_{\min }\ge 3$), such that the modular level of the following nodes is offset as needed. Such colorings can be chosen to respect a prescribed level for  nodes and, a predetermined color for the first node/base pair of the helix

### Proof

The proof is essentially based on case decomposition, and summarized in Fig. 12. We show that, for any *l* and $h_{\min }\ge 3$, there exists a color assignment to the first 3 nodes of the helix, such that the modular level of upcoming nodes is either 0 or 1, so $l'$ can be reached. Moreover, if such a coloring starts with  or , and uses a single  node, then there exists an alternative coloring placing this  node at the opposite modular level, so one of them places their  node at the intended level $\overline{\xi _L}$. Finally, if the first node is set to , then the consistency condition above implies that $l \bmod 2 = \overline{\xi _L}$, so that  nodes are naturally found at an admissible modular level. $\square$

### Theorem 12

Every $(m_{3\bullet },m_{5})$-avoiding target $T$, having $h_{\min }\ge 3$, admits a 2-separated coloring

### Proof

Given *T* a secondary structure as a tree. First, let us remark that helices of *T* can be treated as atomic objects, and compacted into the edges of a *helix tree* *H*, whose edges correspond to helices (sequence of consecutive BP nodes, each BP node has one BP child and no leaf child) in *T*, and whose nodes corresponds to loops in *T*. We distinguish two types of nodes in *H* depending on their corresponding types in *T*:

- *Multiloops in T* *with 2 or 3 BP children in* *T*, *and no leaf. It yields nodes in* *H* *with a degree 3 or 4; (so* $m_{3\bullet }$ *and* $m_{5}$ *do not occur)*
- *Internal/Bulges/Hairpin (IBH) loops in* *T* *delimited by 1 or 2 BPs in* *T*. *They could be empty (e.g. in the hairpin case) or featuring one or multiple leaves (i.e. unpaired nodes) in T*.

*Remark that, while constructing a separated coloring assigning a modular level* $\xi _L$ *to leaves in* *T*,* those two types of nodes in* *H* a*re the only sources of immutable constraints for the coloring:*

- *Any proper coloring of a multiloop in* *T* *features at least one **node, so the levels of BP children need to be set to a level* $\overline{\xi _L}:= \xi _L +1 \bmod 2$;
- *Any IBH loop in* *T* *could feature one leaf within its children, which needs to be set to a modular level* $\xi _L$.

*Conversely, beyond their first BP, helices may be colored with very limited constraints and can be used to* *offset* *multiloops and IBH loops thanks to Lemma* 6.

*We now show formally that any structure* *T* *corresponding to a helix tree* *H* *starting with an initial helix* *I* *can be colored into a 2-separated coloring.*

*Starting at initial level* $l=0$ *and having initial BP color* *c* ($\ne$*if* $\xi _L= 0$*), color the rest of* *I* *as shown in the proof of Lemma *6*, depending on* $\overline{\xi _L}$ *and the type of upcoming loop. We target level *$l'=\overline{\xi _L}$ *after* *I* if *M* *corresponds to a multiloop and level* $l'={\xi _L}$ *after* *I* if *M is an IBH loop, while ensuring that**nodes end up at* $\overline{\xi _L}$ *modular level. It can always be done with Lemma* 6. *The BP children nodes of the loop in* *T* *are then colored in a proper/greedy manner yielding potentially *,  and *nodes prescribed at the start of the next helices. Thus, we iterate the process recursively on the children helices of the loop (if any) knowing the color prescription until the full tree is colored.*

*Since its level cannot be offset, the Root node must be treated as a special case. Indeed, if the Root has at least one leaf/unpaired position in* *T*, *then the modular value 0 is taken by the leaf, so we must have* ${\xi _L}=0$.* Conversely, if the Root in* *T* *supports 3 BP children, then at least one needs to start with a **node, so we must have* ${\xi _L}=1$*. Regardless of this restriction on* ${\xi _L}$*, in both cases the first base pair of each helix (if any) supported by the Root can be properly colored, and helices can be independently colored using the above strategy, ultimately yielding a 2-separated coloring. *$\square$

### Corollary 1

Inverse folding, restricted to instances with $h_{\min } \ge 3$ (containing no isolated base pair and no isolated stacks) is solvable in linear time and space.

It is a direct consequence of Theorem 12 and of the DP scheme introduced in the "Fixed parameter tractable algorithm for modulo-separability" Section. Indeed, for $m=2$, the DP algorithm only needs to be run twice ($\xi _L=0$ and $\xi _L=1$) in linear time/space, to produce a 2-separated coloring whenever such a coloring exists (guaranteed by Theorem 12). The coloring can then be transformed into a design, i.e. a solution to the inverse folding problem. Similarly, uniform modulo separated generation can also be performed in linear expected time and space as long as input instances contain only helices of size 3 or more.

### Corollary 2

Let $T$ be a target structure with $h_{\min } \ge 3$, then the following are equivalent: i) $T$ is designable; ii) $T$ is 2-separable; and iii) $T$ avoids $(m_{3\bullet },m_{5})$.

With this result, the hierarchy of instances collapses as depicted on the left of Fig. 11. A natural follow-up question is whether the bound 3 on the helix length is tight. Indeed, there are non-separable and designable instances with $h_{\min } = 1$ (Proposition 2), but the question remains for $h_{\min } =2$. In Proposition 3 we give a non-separable instance without isolated base pairs, so $h_{\min } =3$ is indeed tight to ensure separability.

## Assessing the relevance of separated sequences towards realistic designs

While the existence of a linear-time algorithm for a reasonable restriction of the inverse folding problem is already notable, its practical relevance may be perceived as hindered by several limitations: our algorithms are only guaranteed to produce design solutions for helices beyond 3 base pairs; proper colorings only allows the design of highly-constrained (multi)loops; and solutions to the base pair inverse folding are not guaranteed to represent good solutions in more realistic energy models, such as the Turner nearest-neighbor model. To assess the potential of separated designs to inform future RNA design methods, we performed computational experiments, using a Python implementation available at:

https://gitlab.inria.fr/amibio/linearbpdesign

### Targets with isolated BPs/stacks are frequently separable

While our algorithm is only guaranteed to produce a design when $h_{\min } \ge 3$, it also produces (guaranteed correct) solutions for input with smaller helices, as long as a separated coloring exists for them. For very small targets, an exhaustive analysis is feasible, consisting of folding/testing the unicity of the MFE folding for all sequences of length $n=12$ (see Fig. 1). Moreover, once a design $w$ is found for a target $T$, it is easy to test if the associated coloring $\chi _{w}$ is separated, and to compute minimal modulus value $m^{\ominus }$ such that $\chi _{w}$ is $m^{\ominus }$ separated. We found that *all of the 8 111 designable targets are also separable*, despite a very large proportion of them featuring isolated stacks and base pairs. Moreover, all designable targets admit separated solutions associated with very small values of the modulus *m* (7 690 for $m=2$, 420 for $m=3$ and $m=1$ only for the empty structure).

**Fig. 13:**
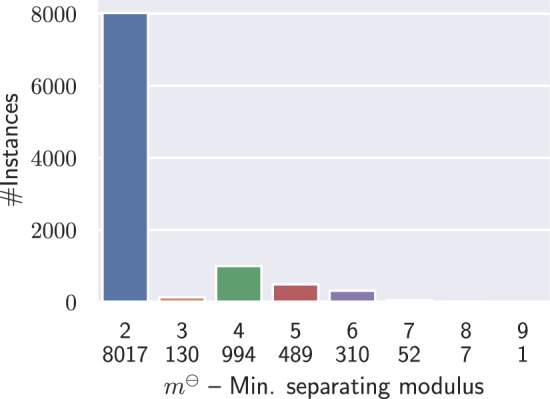
Minimal modulus $m^\ominus$ required to separate 10 000 random targets ($n=100$; $\theta = 3$) featuring $1^{+}$ isolated stack(s). All targets were found to be separable, with $m^\ominus \le 9$

**Fig. 14:**
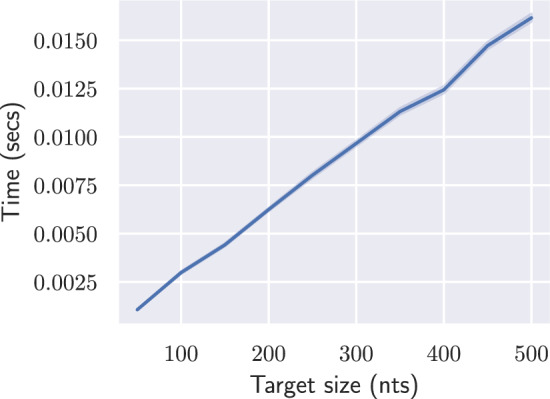
Average runtime of our algorithm (preprocessing + sampling of single instance) for separable instances ($h_{\min }$=3; no $m_{3\bullet }$/$m_{5}$) on a domestic laptop (AMD Ryzen 7 3700U)

To further measure the proportion of separable structures within larger targets featuring isolated stacks, we implemented a uniform random generation algorithm [20]. We produced random target secondary structures of length 100 with a min base pair span of $\theta =3$. Note that our dynamic programming algorithm does not make use of this property as we forbid every alternative base pair with no regard to the distance between the extremities of these base pairs. However, it is realistic to focus our attention on the target structures with $\theta = 3$ relevant in the Turner energy model. We used rejection to produce a synthetic dataset consisting of 10000 targets having at least one helix of size 2 while avoiding $m_{3\bullet }$ and $m_{5}$. For each target $T$, we ran an in-house implementation of the algorithm in the "Fixed parameter tractable algorithm for modulo-separability" section with increasing modulus, to find the minimal modulus $m^{\ominus }$ such that $T$ admits an $m^{\ominus }$ separated coloring. Figure 13 summarizes our results, which we discuss below, while Fig. 14 reports the runtime of our prototype implementation.

Remarkably, all of the 10000 targets in the datasets could be designed using our algorithm, and thus admit a separable coloring. Moreover, roughly three-quarters (80%) of the targets were found to be 2-separable, and less than 1% of the targets required the consideration of values for $m^{\ominus }$ beyond 6. The max value for $m^{\ominus }$ in this dataset was 9, an order of magnitude lower than the sequence length. Clearly, since we have shown the existence of non-separable instances with isolated stacks and no isolated base pair, this observation does not generalize to arbitrary sequence lengths. However, the large size of these counterexamples suggests that the proportion of separable structures, despite ultimately decaying exponentially [19], may remain non-negligible for relevant RNA target sizes.

### Separated designs are promising seeds in the Turner model

We now consider a more realistic setting, where the inverse folding problem is now considered with respect to the Turner nearest-neighbor energy model [22]. To assess the value of a sequence in the Turner model, we introduce a metrics which we call the (signed) *energy distance* $\Delta \Delta G(w,T)$ of a target $T$ to its *most stable distant alternative* for the sequence $w$:

$$\Delta \Delta G(w,T):= \Delta G (w,\alpha _{d^{-}}(w,T)) - \Delta G(w,T),$$

where $\alpha (w,T):= \min \{\Delta G(w,T')\mid |T',T|\ge {d^{-}}\}$ with $\Delta G(w,T)$ the Turner free-energy, $|T,T'|:= |T\,\triangle \, T'|$ denotes the base-pair distance, and ${d^{-}}$ represents the minimum base pair distance to $T$. Both $\Delta G$ and $\alpha _{d^{-}}(w,T)$ can be obtained by appropriate calls to the ViennaRNA package [1], namely RNAeval and RNAsubopts, using max energy distance parameter $E=5$ (so our estimation of $\Delta \Delta G(w,T)$ is bounded by 5).

A positive energy distance confirms that $w$ is a solution to the Turner version of inverse folding, and dominates its competitors by $\Delta \Delta G(w,T)\,\text {kcal.mol}^{-1}$. Meanwhile, a negative energy distance indicates that the target $T$ is dominated by some alternative structure, having $\Delta \Delta G(w,T)\,\text {kcal.mol}^{-1}$ lower free-energy than the target.

We consider four strategies for generating sequences:

1. The *compatible* model uniformly generates random sequences compatible with the target (A for unpaired positions; AU, UA, GC or CG for base pairs);
2. The separated model uses the sampler described in the "Random generation of *m*-separated sequences" section to generate sequences that are 2-separated and proper;
3. The relaxed, sometimes also called the unproper model, generates sequences that are 2-separated, but not necessarily proper, assigning uniform random pairs to the base pairs of a multiloop. This model enables a heuristic extension of our algorithms supporting multiloops of arbitrary degrees. Indeed, the local refolding (see Fig. 4), induced by non-proper sequences in the BP model, are either unrealistic or outright impossible in the Turner energy model;
4. Finally, we used as a baseline InfoRNA [4], a seminal method relying on local search to solve the inverse folding in the Turner model. At its core, a deterministic seeding algorithm uses dynamic programming to find the single sequence that achieves minimum free energy for the target.

To ensure a fair comparison, all $\Delta \Delta G$ calculations were performed using the Turner99 model, since it is the only one readily supported by InfoRNA [4].

#### Separated sequences outperform compatible sequences

We first asked a basic question: *Are separated sequences better candidates for design in the Turner model than sequences compatible with the target?* The answer is not obvious since separated sequences are only guaranteed to represent designs for the BP max. model. We considered instances of size $n=100$ admitting a solution to $\text{{Inverse-Folding}}_{\textbf {BP}}$ ($\theta = 3$; no $m_{3\bullet }$/$m_{5}$; $h_{\min }\ge 3$). We generated 1000 random targets and, for each target, sampled a single sequence using each of the 3 strategies above and computed the energy distance.

**Fig. 15:**
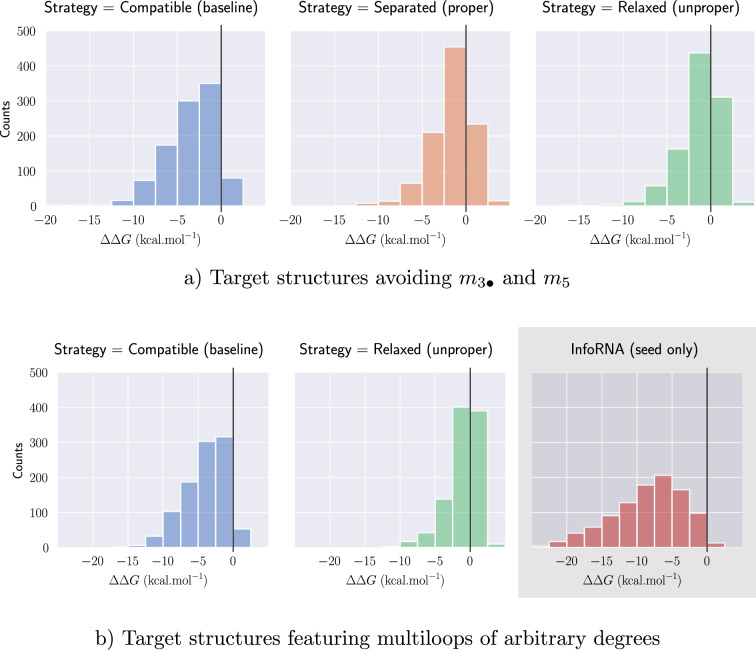
Comparison of compatible (baseline), separated, relaxed models and InfoRNA [4] for targets having $n=100, \theta =3, h_{\min } =3$. For energy distance parameters, we took ${d^{-}}=3$. Free-energies and MFEs were computed using the Turner99 model to ensure a fair comparison with InfoRNA

The results, summarized in Fig. 15 top suggest that separated sequences represent a substantial improvement over merely compatible sequences. Indeed, while 10% of compatible sequences ended up being good design candidates ($\Delta \Delta G>0$), the proportion of successful designs increases to approximately one-third (35%) for separated sequences, and further to 43% for relaxed design. A similar trend can be observed for the average $\Delta \Delta G$ (distance to the first alternative/competitor) among successful designs, being of 0.79/0.98/1.06 kcal.$\hbox {mol}^{-1}$ in the compatible, separated and relaxed models respectively.

The good behavior of the relaxed model, which was mostly introduced to overcome unrealistic limitations on multiloops, is surprising. Intuitively, the proper condition allows to rule out local alternatives caused by the creation of unwanted alternative base pairs, within a loop as seen in Figs. 2 and 3C. Such base pairs involve positions that are either very close, or lead to the creation of additional bulges/internal loops, both of which are typically penalized (or even outright forbidden) by the Turner model, so the associated structures are not competitive in realistic models. However, this argument is only partially convincing, since the intricacies of the Turner model may cause unforeseen – radically different – structures, possibly with fewer base pairs, to become competitive to the target. A fully satisfactory explanation of the phenomenon thus remains to be formulated.

#### Relaxed sequences enable designs for higher degrees multiloops

We also tested the capacity of the relaxed model to generate solutions for multiloops of higher degrees, noting that the avoidance of $m_{3\bullet }$ and $m_{5}$ restricts the maximum degree of a multiloop to 4. We used the above-mentioned generation algorithm to generate uniform design targets of size $n=100$, featuring at least one (but frequently many) occurrence of $m_{3\bullet }$ and $m_{5}$. As shown in Fig. 15 bottom, compatible sequences are again substantially outperformed by the relaxed separated model in this setting, with 40% of the separated/non-proper sequences representing successful designs ($\Delta \Delta G>0$; vs 5.3% for compatible sequences), and are on average 0.87 kcal.$\hbox {mol}^{-1}$ more stable than their best competitor.

Conversely, InfoRNA rarely generates sequences that fold optimally according to the Turner energy model. Namely, only 1.3% of the sequences returned by InfoRNA initialization strategy are optimal for their target. Instead, the MFE structure of InfoRNA sequence typically differs from its target, and is much more stable by 8.65 kcal.$\hbox {mol}^{-1}$ on average (vs 2.23 kcal.$\hbox {mol}^{-1}$ for unproper sequences).

This disappointing behavior of InfoRNA was unexpected an pronounced, as even compatible random sequences with A at unpaired positions (i.e. baseline sequences) appear to represent better starting points in the Turner energy model. We also observed that InfoRNA often returns sequences where the target structure is a strict subset of the MFE. This suggests that such sequences would probably substantially benefit from forcing A on unpaired positions, but the design of such a mitigation strategy exceeds the scope of this work.

### Using multidimensional Boltzmann sampling to control GC content

Targeting a realistic G+C content (GC%) is a traditional secondary objective of inverse folding [12]. In particular, it is generally believed that solution sequences featuring artificially high GC% ($\gg 50\%$) are somewhat easier to find computationally yet may suffer from slow kinetics due to the transient formation of alternative stable helices, delaying convergence to the thermodynamic equilibrium.

To control the GC% of *m*-separated designs produced by our random generation algorithm (cf "Random generation of *m*-separated sequences" section), we use multidimensional Boltzmann sampling, a technique introduced in the context of enumerative combinatorics [23, 24], and more recently adapted to efficiently constrain stochastic sampling within classified dynamic programming [12, 15, 25, 26]. Its core idea is to induce a Boltzmann distribution for the emission probabilities, such that:

$$\mathbb {P}(w\mid T,\pi _{\textsf {GC}}) = \frac{e^{\pi _{\textsf {GC}}\cdot \#\textsf {GC}(w)}}{\mathcal {Z}_{\pi _{\textsf {GC}}}}, \quad \mathcal {Z}_{\pi _{\textsf {GC}}}:= \sum _{w' \text { { m}-sep. for }T} e^{\pi _{\textsf {GC}}\cdot \#\textsf {GC}(w')},$$

using a weight $\pi _{\textsf {GC}}\in \mathbb {R}$ whose value can be used to control the expected GC% of generated sequences. Generating in such a distribution can be achieved through a simple adaptation of the random generation algorithm from the "Random generation of *m*-separated sequences" section, to incorporate a multiplicative weight $e^{2\pi _{\textsf {GC}}}$ anytime a G$\cdot$ C or C$\cdot$ G content is chosen for a given base pair. Then, a rejection strategy keeps only those sequences having desired GC%, resulting in a random generation algorithm with guaranteed uniformity at fixed GC%. Using a binary search for a suitable value of $\pi _{\textsf {GC}}$, the associated average-case time complexity then increases as $\Theta (n\sqrt{n})$ under mild concentration assumptions (i.e. asymptotic convergence of GC% to a Normal distribution).

**Fig. 16:**
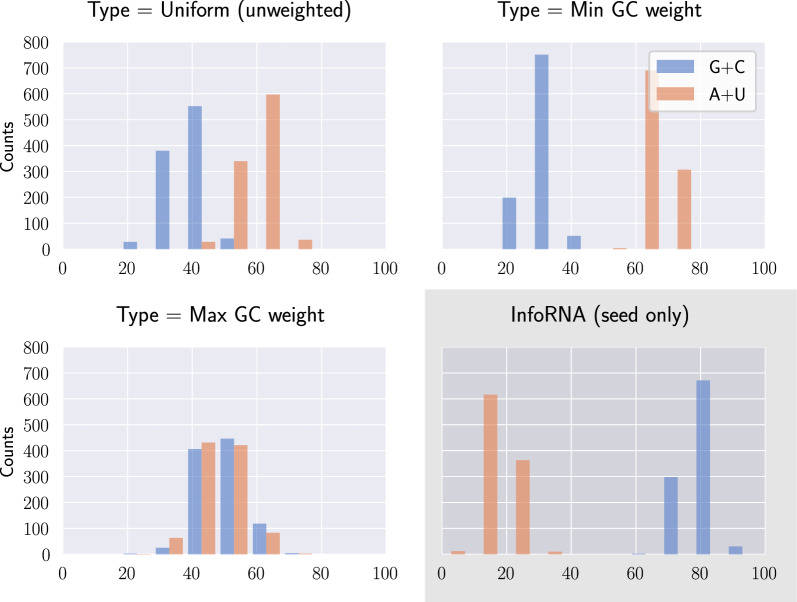
Modulating the GC% of random 2-separated designs. *Natural* GC% distribution (Top Left) observed among random 2-separated, uniformly-distributed, sequences for random targets (1 structure per target). Using a Boltzmann distribution $\mathbb {P}(w) \propto e^{\pi _{\textsf {GC}}\cdot \#\textsf {GC}(w)}$ instead allows a fine control over the GC%. Moreover, using extreme values of $\pi _{\textsf {GC}}$, the GC% can either be minimized (Top Right), or maximized (Bottom Left). We display the distributions for InfoRNA, an alternative design methodology (Bottom Right)

We generated 1 000 uniform random separable structures ($m_{3\bullet }$/$m_{5}$-free; $h_{\min } =3$) of length 100nts. For each structure, we produced a single *m*-separated design ($\bmod$ 2-separated sequences), initially in the uniform distribution $\pi _{\textsf {GC}} = 0$ to determine the typical GC% distribution. The results, summarized in Fig. 16 (Top.Left), show that a low average GC% of 40% (40% median) can be consistently reached (5.4% std). Unsurprisingly, extreme GC% values are difficult to reach, both due to the assignment of A to unpaired positions (37.5% of total positions), and the necessity to alternate G$\cdot$ C/C$\cdot$ G and A$\cdot$ U/U$\cdot$ A within multiloops.

We then reprocessed the same structure dataset, this time using a numerical iteration to determine values of $\pi _{\textsf {GC}}$ which minimize GC% while avoiding numerical underflows ($-59\le \pi _{\textsf {GC}} \le -34$). Figure 16 (Top.Right) showcases the resulting GC% distribution, which is tightly concentrated around 32.5% (3.8 std).

Finally, the GC% can be pushed by setting $\pi _{\textsf {GC}}$ to its maximum while avoiding numerical overflows ($18\le \pi _{\textsf {GC}} \le 39$). Again, we observe, in Fig. 16 (Bottom.Left), a relatively tight concentration around the mean value of 51% (6.8 std), approximately equating the GC% and AU%. In comparison, the initialization strategy of InfoRNA gives an unrealistically high GC% of around 82% (4.2 std), as can be seen on Fig. 16 (Bottom.Right).

Overall, this study confirms that modulo 2 separated sequences, despite being a strict subset of all designable sequences, represent a sufficiently rich family to imprint further constraints, as demonstrated here by our modulation of the GC%. Future work may consider the utilization of such sequences as reasonable starting points (*aka* seeds) for design heuristics targeting the Turner energy model [1].

## Conclusion

Adapting a coloring perspective initially introduced by Halès et al. [17], we have shown that the inverse folding problem can be solved in linear time for all target secondary structures having minimum helix length equal to 3. Towards that main result, we have established the existence of designable, yet non-separable, instances of inverse folding, and the NP-hardness of finding a separable design in the initial sense of Halès et al. We have also introduced concrete algorithms for the problem of finding a *m* modulo-separated coloring, which we have shown to be NP-hard yet FPT-solvable for *m*. Already for $m=2$, the scope of our algorithms encompasses all targets without isolated base pairs and stacks, but also extends much beyond, in a way that remains to be fully characterized. Beyond base pair maximization, modulo-separated sequences may also represent a solid foundation towards concrete design methodologies. Namely, we have empirically observed that, for the Turner energy model, separated sequences tend to represent better design candidates than merely compatible sequences, and that the limitations on loop degrees (intrinsic to the BP maximization model) can be overcome by relaxing our design model while retaining substantial performances. Moreover, we have showed that *m*-separated sequences offer sufficient diversity to modulate the GC content of produced sequences.

Future work should focus on how much of designable sequences are covered by sequences obtained with (modulo)-separated colorings. More importantly, does the space of (modulo)-separated colorings always/often contains designs with respect to the nearest-neighborhood Turner energy model? Even if it unlikely to hold unconditionally, it is plausible that some extensions of separability and *m*-separability will achieve theoretical and practical solutions for inverse folding in more general energy models. As a first step, separability in a stacking energy model seems a relevant goal, even if less ambitious than the Turner nearest-neighbor model. It would probably require to go beyond the current coloring formalism, and motivate the introduction of more general notions of defects to capture imbalance at the level of dinucleotides compositions. Finally, extensions of this work may explore generalizations of the notion of (*m*-)separability, possibly in combination with further constraints-based filters, to directly address *real world* design scenarios. Towards that goal, we have introduced the concept of biseparability to enable a joint presence of $\textsf {A}$s and $\textsf {C}$s in unpaired positions, and used the produced sequence as a starting point (*aka* seed) in the context of various popular heuristics, leading to improved performances [27].

## Data Availability

No datasets were generated or analysed during the current study.

## Footnotes

1 Base pairs may also be leaves of the tree when involving consecutive positions, which happens rarely in practice. We thus qualify as *internal node* any node in bijection with a base pair.
